# *Oenopia shirkuhensis* sp. nov. (Coleoptera, Coccinellidae) from Iran mimicking *Adalia bipunctata*

**DOI:** 10.3897/zookeys.915.46390

**Published:** 2020-02-24

**Authors:** Mehdi Zare Khormizi, Oldřich Nedvěd

**Affiliations:** 1 Yazd Provincial Office of Department of Environment, Yazd, Iran Yazd Provincial Office of Department of Environment Yazd Iran; 2 Faculty of Science, University of South Bohemia, České Budějovice, Czech Republic University of South Bohemia České Budějovice Czech Republic; 3 Biology Centre, Czech Academy of Sciences, České Budějovice, Czech Republic Biology Centre, Czech Academy of Sciences České Budějovice Czech Republic

**Keywords:** ladybug, Müllerian mimicry, new species

## Abstract

*Oenopia shirkuhensis***sp. nov.** (Coleoptera, Coccinellidae) is described and illustrated. It was found in the mountains around Shirkooh mountain, Yazd province, and in the Kukhbenan Mountains, Kerman province, Iran. It is similar to a common ladybird *Adalia bipunctata* by the colour pattern on elytra. Congeneric species occurring in Iran, *O. conglobata* and partly *O. oncina* are illustrated for comparison, and an identification key is provided.

## Introduction

Coccinellidae is a diverse beetle family well known and appreciated by the wide public mainly due to their attractive colour patterns with spots and stripes. The tribe Coccinellini, which includes now about 94 valid genera (Nedvěd 2015; Escalona et al. 2017), especially shows high interspecific and also intraspecific variability in elytral colour patterns. Groups of similarly coloured species of ladybirds seem to form Müllerian mimicry rings, where certain aposematic patterns are shared among sympatric species (Brakefield 1985). Polymorphic ladybird species, such as *Adalia bipunctata* (Linnaeus, 1758) and *A. decempunctata* (Linnaeus, 1758), have several forms that are members of different mimicry species rings.

*Oenopia* Mulsant, 1850 is a relatively species-rich genus of ladybirds with 21 species occurring in the Palearctic and Oriental regions (Iablokoff-Khnzorian 1982). There have been three species of *Oenopia* reported so far from Iran: *O. conglobata* (Linnaeus, 1758), *O. montana* Savoyskaya, 1969, and *O. oncina* (Olivier, 1808) (Abdolahi Mesbah et al. 2016).

Species of the genus *Oenopia* have a medium-sized body (2.5–6 mm), which is broadly oval to oval (length/width 1.25–1.5) and moderately convex. Antennae are as long as the width of the frons between eyes. Prosternal process bears carinae that do not reach anterior margin of prosternum. Shallow fovea in the middle of the anterior margin of mesoventrite bears the prosternal process (Fig. 5). Scutellum is 8–15×, epipleuron 6–12× narrower than body in their widest positions. Episterna and epimera of metathorax are black. Postcoxal line of the abdominal ventrite 1 is incomplete, either in the form of quarter of circle or divided into two parts, one parallel to the hind margin and one oblique. Tibial spurs are small, tarsal claws have basal tooth (except in *O. montana*). Distal part of penis is strongly narrowed, penis guide is wide, often bifurcated and with the tip bent upwards. The species are arboreal, aphidophagous.

We describe and illustrate a new species of *Oenopia* from high mountains of central Iran. It is compared to the common *O. conglobata* and to the unrelated *A. bipunctata* which have similar elytral colour pattern as the new species.

## Material and methods

Morphological terminology follows Ślipiński (2007). Photographs were taken using a Nikon SMZ 1500 stereo microscope with Lumenera digital camera and QuickPhoto software. Composite images with deep focus were generated using Zerene stacker. Genitalia were dissected, boiled in 10% KOH, washed in water, alcohol, acetone, and xylene, then submerged to artificial resin on a microscopic slide. Photos of *Oenopia conglobata* were taken with Nikon D850 and handmade macro lens and processed with Zerene stacker.

The following measurements were taken: **BL** = total body length from clypeus to apex of elytra; **BW** = body width across both elytra at widest point; **HW** = head width including eyes; **PL** = pronotal length, from middle of anterior margin to base of pronotum; **PW** = pronotal width at widest point; **EL** = elytral length from the base including the scutellum to apex of the elytra.

## Results

### Oenopia shirkuhensis

Taxon classificationAnimaliaColeopteraCoccinellidae

Zare Khormizi & Nedvěd sp. nov.

E801591D-3B9E-599C-811E-9F24FC35CAF4

http://zoobank.org/69FF66FE-C039-496B-BD62-F50A564F9A51

#### Type material.

***Holotype.*** Male, Sorgan valley, Shirkooh mountain, Seikhalishah parish Dehbala village, Taft region, Yazd province, Iran, 31°37.50'N, 54°05.68'E, 2960 m a.s.l. 16. v. 2014. On *Rosa canina*. Lgt. Mehdi Zare Khormizi, Fig. 2.

**Figure 1.**
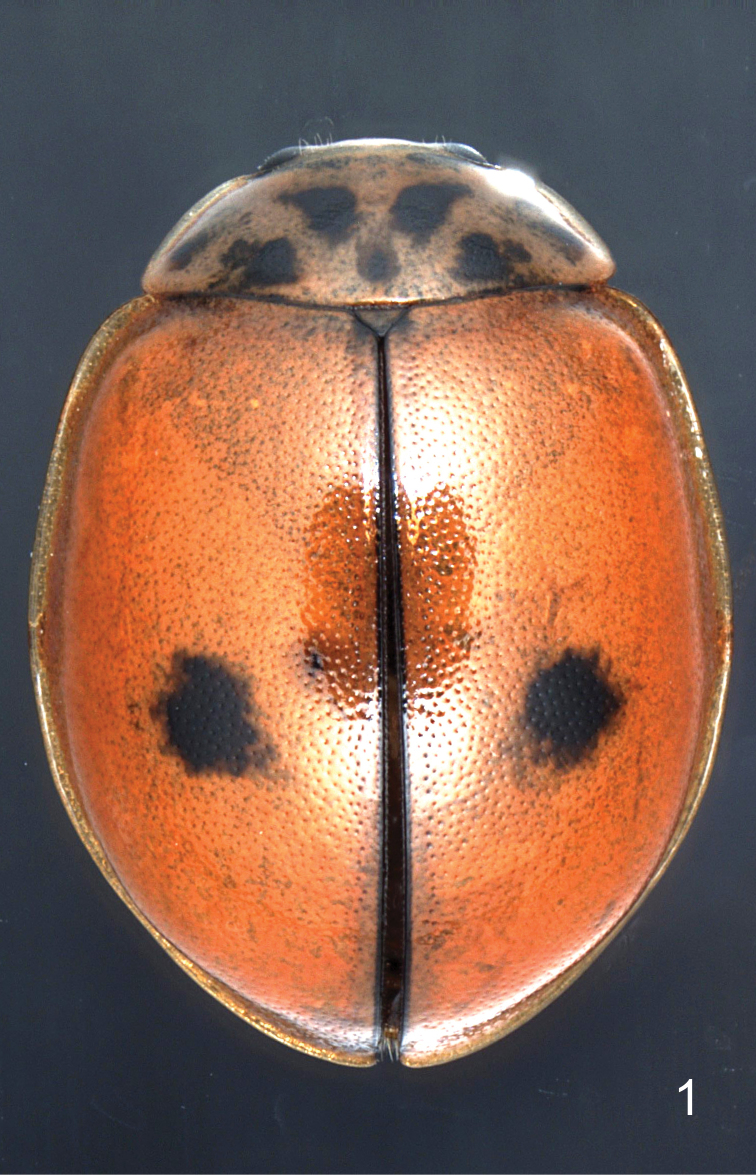
*Oenopia shirkuhensis* sp. nov., paratype 1 (female), dorsal view.

***Paratypes.*** 5 males and 4 females in the same location and the same other data; 2 females at the beginning of the Najib valley, 31°37.60'N, 54°05.38'E, 3130 m a.s.l., 3 males and 2 females at the end of the Sorgan valley, 31°37.65'N, 54°05.35'E, 3155 m a.s.l., other data same. Figs 1, 3.

**Figures 2–10.**
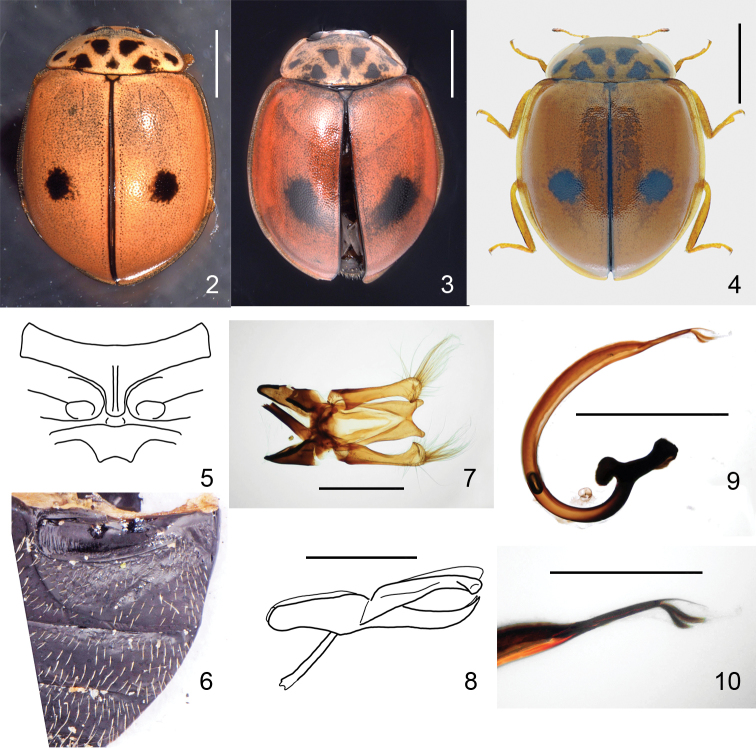
*Oenopia shirkuhensis* sp. nov. **2** holotype (male), dorsal view **3** paratype 3 (male), dorsal view. Notice elytral spots extended towards the suture and slightly backward **4** individual from Kerman province, collected by Yu. Skrylnik, photographed by Alexander Slutsky, dorsal view **5** prosternum and mesosternum. Prosternal carinae extending over more than half of the prosternum length. Fovea in the front margin of mesosternum is shallow **6** paratype 2, first abdominal ventrite, left side. Ventrites completely black. Abdominal postcoxal line bifurcated with the main branch parallel to the posterior margin of the ventrite 1, not reaching lateral margin, secondary branch oblique (45°) separated from the main branch and from the coxal fovea **7** holotype, tegmen, outer view. The tips longer than wide, parabolic shape between the two tips less than two times wide as deep **8** holotype, tegmen, lateral view. The tips are inclined about 45° from the plane of penis guide main body. Parameres as long as the penis guide **9** holotype, penis **10** holotype, tip of penis. Scale bars: 1 mm (**2, 3, 4, 9**); 0.5 mm (**7, 8, 10**).

The holotype and one paratype of the new species will be deposited in the National Museum, Prague, Czechia. Five paratypes will be deposited at the University of South Bohemia, České Budějovice, Czechia. All specimens are in alcohol, but genitalia are on microscope glass in resin. Ten paratypes will be deposited in the collection of the first author, in Yazd, Iran. All are frozen.

#### Other material examined.

Iran, Kerman province, 5.5 km E of Sekonj village, Kukhbenan Mountains, at 30°00.00'N, 57°28.53'E, 2540 m a.s.l., 28. vi. 2014, lgt. Skrylnik Yu., 1 individual, determined based on the photograph made by Alexander Slutsky, Fig. 4.

#### Etymology.

Adjective referring to the place of sampling, on the slopes of Shirkooh mountain (which means Lion mountain).

#### Diagnosis.

**Figures 11–17.**
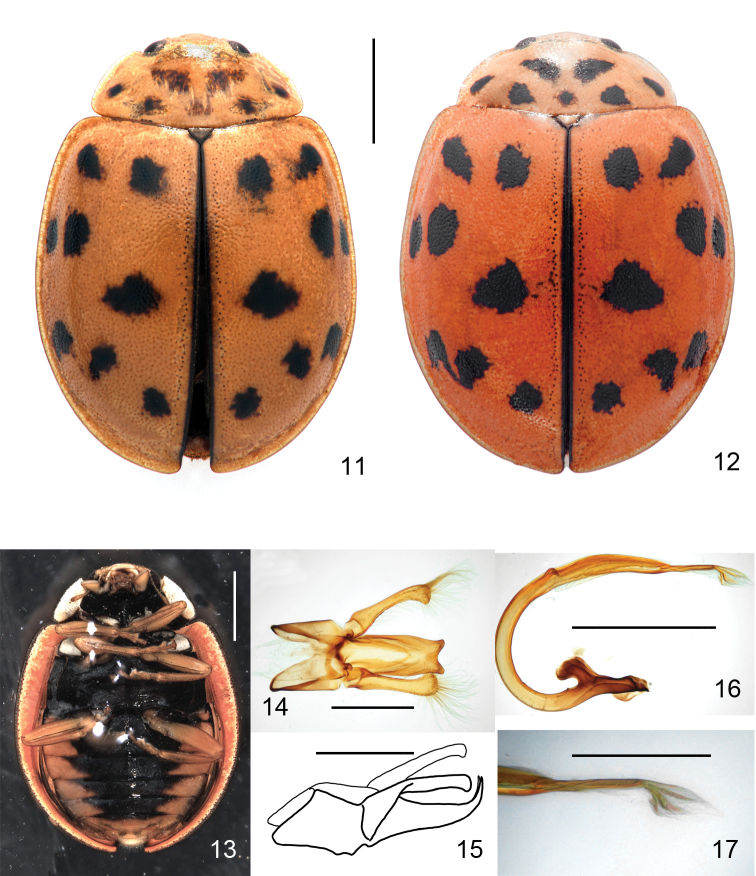
*Oenopia conglobata***11** from Yazd province, Iran, yellowish form. Dorsal view **12** from Yazd province, Iran, rose form. Dorsal view **13** from Chaharmahal and Bakhtiary province, Iran. Ventral view. Abdominal ventrites with large triangular yellow-brown margins **14** tegmen, outer view. The tips of penis guide shorter than wide, parabolic shape between the two tips more than three times wide as deep **15** tegmen, lateral view. The tips are inclined about 80° from the plane of penis guide main body. Parameres slightly shorter than the penis guide **16** penis **17** tip of penis. Scale bars: 1 mm (**11, 13, 16**); 0.5 mm (**14, 15, 17**).

This species can be separated from its congeners by the colour pattern of elytra, which is orange-red with only one pair of rounded black spots lying slightly behind the half length of the elytra, either in the middle between suture and lateral margin (Fig. 2) or extended to the suture (Fig. 3), which bears a narrow black stripe. In the closest relative, the elytra has 16 spots (Figs 11, 12). Abdominal ventrites are completely black (Fig. 6), while they have yellow margins in some congeners (Fig. 13). The two tips of penis guide are longer than wide and the parabolic shape between them, in outer view, is less than two times as wide as deep (Fig. 7). The tips in lateral view are inclined about 45° from the plane of main body (Fig. 8). Parameres are as long as the penis guide. In *Oenopia conglobata*, the tips of penis guide are shorter than wide, and the parabolic shape between the two tips are more than three times as wide as deep (Fig. 14), the tips are inclined about 80° from the plane of penis guide main body (Fig. 15), and the parameres are slightly shorter than the guide.

#### Description.

***Body*** outline (Figs 1–4) broad oval, 1.35× as long as wide, broadest behind middle of elytra. Dorsum convex and glabrous, except few hairs on head near eyes. Interocular distance 2.7× as wide as eye. Pronotum with fine punctures. Elytra with large shallow punctures, separated by 1–2 diameters, interspaces between punctures smooth, shiny.

***Head*** creamy white. ***Pronotum*** creamy white with seven black spots forming triple VVV, with two pairs of additional small spots lateral to large mediolateral spots. ***Elytra*** orange-red, shiny, with one pair of rounded black spots lying slightly behind the half length of elytra (see Table 1), in the middle between suture and lateral margin or eliptical, extended to the suture, which bears a narrow (quarter of width of scutellum) black stripe. Pronotum and elytra with explanate lateral margins transparent.

Prosternal intercoxal process (Fig. 5) narrow, with a pair of carinae extending over two-thirds of the prosternum length. Ventral side of thorax black, mesepimeron white with narrow black margins. Epipleuron 8× narrower than body width. Scutellum 13× narrower than body width (Fig. 1).

***Abdomen*** with ventrites completely black. Abdominal postcoxal line (Fig. 6) bifurcated with the main branch parallel to the posterior margin of the ventrite 1, almost reaching lateral margin, secondary branch oblique (45°) separated from the main branch and from the coxal cavity.

***Legs*** orange-brown, coxae black, fore coxae with white spot. Tarsal claws with basal tooth.

Male ***genitalia*** (Figs 7–10) with penis guide of tegmen in outer view (Fig. 7) bifurcated; penis guide slightly constricted before tips. Tips longer than wide, parabolic shape between tips less than two-times as wide as deep. Parameres as long as penis guide, clavate, truncate at apices with dense and long hairs. Penis guide in lateral view (Fig. 8) gradually narrowed. Tips in lateral view inclined at about 45° from main body plane. Penis (Fig. 9) with well-developed basal capsule with inner and outer arms subequal; penis apex strongly narrowed, then enlarged to wide soft tip (Fig. 10).

***Body measurements***: in mm; first value belongs to holotype, others to paratypes (smallest, largest, and most beautiful, i.e. female in Fig. 1, of the paratypes); **BL** = 3.87, 3.76, 4.31, 4.05; **BW** = 2.89, 2.87, 3.35, 2.92; **HW** = 1.01, 0.96, 1.12, 1.03; **PL** = 0.88, 0.74, 0.88, 0.80; **PW** = 1.90, 1.92, 2.21, 2.08; **EL** = 2.79, 2.87, 3.21, 3.16.

##### Key to the Iranian *Oenopia*

**Table:** 

1	Main elytral colour light (beige, pink, or orange) with dark spots and lines. Pronotum light with separate dark spots	**2**
–	Elytra black with light spots or at least light fore margin. Pronotum black with light anterior and lateral margins	***O. oncina***
2	Elytra beige, pink, or orange to red, with rounded dark spots at least in posterior half. Mesepimeron white with narrow black margins; metaepimera and metepisterna dark. Tarsal claw with basal tooth	**3**
–	Elytra beige with small dark dots in anterior half and irregular lines in posterior half. Mesepimera, metaepimera, and metepisterna pale. Tarsal claw without tooth	***O. montana***
3	Elytra with up to 16 spots (pattern 2+2+1+3), either black or brown with lighter center. Penis guide with tip curved upwards in right angle	***O. conglobata***
–	Elytra with one pair of black spots behind center. Penis guide with tip slightly curved upwards at 45° angle	***O. shirkuhensis***

## Discussion

**Figures 18, 19.**
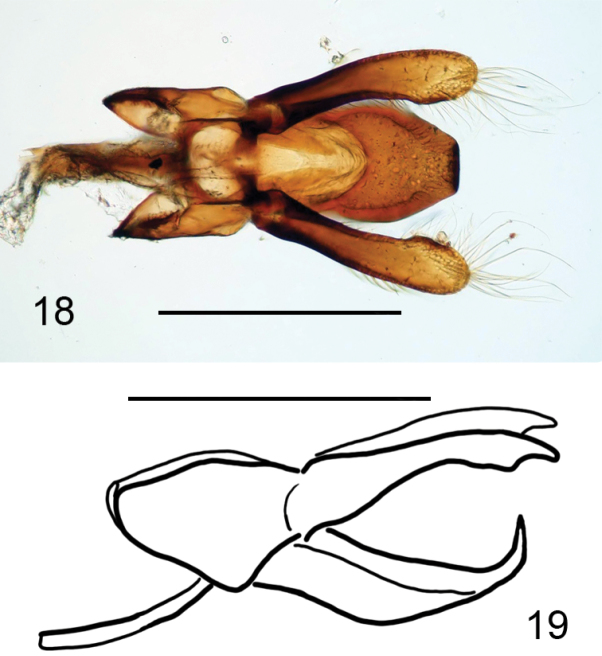
*Oenopia oncina***18** tegmen, outer view. Single tip of penis guide pointed towards the viewer **19** tegmen, lateral view. The tip is inclined more than 90° from the plane of penis guide main body. Parameres slightly longer than the penis guide. Scale bars: 0.5 mm.

The main morphological characteristics of the new species are very similar to the common and widespread *Oenopia conglobata* (Figs 11–17). The colour pattern of the new species superficially resembles *A. bipunctata* but may be derived from the pattern of *O. conglobata*, if only the pair of the largest spots is retained. The position of these spots on the elytra is more similar between the two *Oenopia* species than between *O. shirkuhensis* and *A. bipunctata* (Table 1). *Oenopia conglobata* varies in background colour (Figs 11, 12). This includes *O. conglobata contaminata* (Ménétriés, 1849) with pale pink (dominant) and pale red (less common) individuals (Zare Khormizi et al. 2016). Other Asian species of *Oenopia* possess much different elytral pattern and shape of genitalia (Figs 18, 19; Iablokoff-Khnzorian 1982). Thus, we hypothesize that *O. conglobata* and *O. shirkuhensis* are closely related species and that *O. shirkuhensis* speciated in the isolated environment of high mountains surrounded by deserts. We extracted DNA from two paratypes, but it was degraded, and no PCR product needed for identification and estimation of evolutionary distance between the two species was obtained.

**Table 1.** Comparative body and pattern measurements of *Oenopia shirkuhensis*, related *O. conglobata* and visually similar *A. bipunctata*. A: body length/width ratio; B: position of centre of the main elytral spot as relative longitudinal distance from the front margin of elytra; C: distance of the centres of the main elytral spots as transversal distance relative to the width of elytra.

	A	B	C
*O. shirkuhensis* sp. nov.	1.32–1.39	52–53%	50–53%
*O. conglobata*	1.30–1.49	50–54%	38–46%
*A. bipunctata*	1.37–1.50	46–48%	59–63%

The new species was the only coccinellid species found at that high elevation (2960–3155 m a.s.l.) on the slopes of Shirkooh mountain (4055 m high). The nearest locality with other ladybirds was at the hillside at the beginning of the Sorgan valley (31°37.30'N, 54°05.97'E, 2760 m a.s.l.) There, the first author found four species of ladybirds on asafoetida plants (*Ferula assa-foetida* L., 1753): *Hippodamia variegata* (Goeze, 1777) (2 exx.), *Coccinella septempunctata* (Linnaeus, 1758) (1 ex.), *Oenopia oncina* (1 ex.), *Scymnus subvillosus* (Goeze, 1777) (1 ex.). Neither *O. conglobata* nor *A. bipunctata* were found nearby.

The striking similarity of *O. shirkuhensis* to A. bipunctata f. typica could be explained by Müllerian mimicry. *O. shirkuhensis* has 1) orange-red colour background and 2) only two black spots on elytra in similar position as *A. bipunctata* (but see exact positioning in Table 1). Both characters are apparently apomorphies, not found in other *Oenopia* species, except *O. conglobata*. However, living in an isolated alpine environment without presence of the putative model (*A. bipunctata*) brings to question Müllerian mimicry as a mechanism for the evolution of the colour pattern. As an opposite possible example of Müllerian mimicry, we (Nedvěd et al. in press) found individuals of A. bipunctata f. fasciatopunctata, with pink background unusual for the genus *Adalia*, living together with typical pink *O. conglobata* in Chaharmahal and Bakhtiary province. The new species possesses very light colouration even though it is advantageous to many insects to be dark in alpine habitats and thus use sunshine to heat up the body (e.g. compare with Maclean et al. 2016). Similarly, *O. montana* from mountains of Kazakhstan to Kirghizstan and *O. signatella* (Mulsant, 1850) from the Himalayas have very light colouration (Iablokoff-Khnzorian 1982).

Shirkooh mountain is one of the central mountains of Iran. According to the latest studies on Shirkooh mountain vegetation, 610 plant species have been identified and listed in the region (Irannezhad 2012), about 70% of the flora of Yazd province. This high diversity relative to the small area within the large province indicates the high plant species richness of the region. Of the 610 species, 91 have a special conservation value and are listed on the Red List of plants of Iran (Mathew et al. 2000). Thus, we expect also high number of invertebrates, including undescribed ones. The second locality of the new species in Kerman province is located 150 km from Shirkooh. Desert separates the two localities.

## Supplementary Material

XML Treatment for Oenopia shirkuhensis
